# Prediction of Computer Vision Syndrome in Health Personnel by Means of Genetic Algorithms and Binary Regression Trees †

**DOI:** 10.3390/s19122800

**Published:** 2019-06-22

**Authors:** Eva María Artime Ríos, Fernando Sánchez Lasheras, Ana Suárez Sánchez, Francisco J. Iglesias-Rodríguez, María del Mar Seguí Crespo

**Affiliations:** 1 Central University Hospital of Asturias, 33011 Oviedo, Spain; 2 Department of Mathematics, University of Oviedo, 33007 Oviedo, Spain; sanchezfernando@uniovi.es; 3 Department of Business Administration, University of Oviedo, 33004 Oviedo, Spain; suarezana@uniovi.es (A.S.S.); fjiglesias@uniovi.es (F.J.I.-R.); 4 Department of Optics, Pharmacology and Anatomy, University of Alicante, 03690 Alicante, Spain; mm.segui@ua.es

**Keywords:** genetic algorithms, regression tree, computer vision syndrome, health personnel, occupational health

## Abstract

One of the major consequences of the digital revolution has been the increase in the use of electronic devices in health services. Despite their remarkable advantages, though, the use of computers and other visual display terminals for a prolonged time may have negative effects on vision, leading to a greater risk of Computer Vision Syndrome (CVS) among their users. In this study, the importance of ocular and visual symptoms related to CVS was evaluated, and the factors associated with CVS were studied, with the help of an algorithm based on regression trees and genetic algorithms. The performance of this proposed model was also tested to check its ability to predict how prone a worker is to suffering from CVS. The findings of the present research confirm a high prevalence of CVS in healthcare workers, and associate CVS with a longer duration of occupation and higher daily computer usage.

## 1. Introduction

Research in the field of human–computer interaction usually observes the ways in which humans interact with computers, but the interaction of the computer with the human lies within this field as well, introducing another perspective that increasingly needs attention. The introduction of information and communication technologies (ICT), as well as the use of devices like computers, smartphones, and laptops with visual display terminals (VDT), has brought new forms of work, management, and organization into the professional world, implying great transformations and changes in business organization.

The VI European Working Condition Survey has reported that the use of ICT is most widespread in the service sectors—mainly in financial services, but also in public administration, education, and health. In terms of occupation, the most frequent users are managers, professionals, and technicians. It has also been pointed out that the number of workers using ICT devices at least one-quarter of the time at work has risen from 36% in 2005 to 57% in 2015 [1].

Together with the positive changes, human–computer interaction poses several risks for the health of workers. In particular, VDT are practically indispensable nowadays in the computer–human interface, but this essential element may be responsible for diverse ocular and visual disorders. VDT users typically complain of various types of ocular ailments, which include eyestrain, eye fatigue, burning sensations, irritation, redness, blurred vision, and dry eyes, among others [2]. The term “Computer Vision Syndrome” (CVS) is widely used in medical science to refer to the condition of a person experiencing one or more of these ocular complaints as a result of operating a computer and looking at a VDT [2,3,4,5].

CVS is also referred to in existing literature as asthenopia, visual fatigue, eyestrain, visual strain, or ocular symptoms. Various prevalence rates of CVS have been reported in different studies, ranging from less than 20% to more than 80% [6,7,8,9,10,11]. When comparing the results of such studies in different populations, the limitations include differences in the characteristics of the samples, methodologies, and the instruments used for data collection. 

A long duration of computer usage, a reduced number of breaks and rest periods, and prolonged years of use are risk factors for CVS. Increased time of use of VDTs may affect the tear film and the ocular surface, because during computer work the frequency of blinking decreases, with a consequent increase in the evaporation of the tear film that compromises the good condition of the ocular surface [12,13,14,15]. Environmental factors that have been pointed out as possible causes of eye symptoms include bad quality of the indoor air, high room temperature, low relative room humidity, poor lighting conditions, the presence of glare, screen brightness, or improper design of the workstation [4,16]. Two instruments to measure this syndrome [17,18] have been developed and validated recently in Spain. For years, there have been numerous studies that have used patient-reported outcome (PRO) questionnaires to measure the quality of life in relation to visual function and the severity of symptoms in patients, to be used later for diagnostic and therapeutic purposes.

Digital technologies brought electronic health records (EHR) into the National Health System, and healthcare workers became users of VDTs. To our knowledge, few scientific studies have been performed to date that assess the effects of the exposure to VDTs on the visual health status of health personnel. One study in Canada [19] included a sample of radiologists, and estimated the prevalence of CVS at 36%; being a woman, young, working longer work days, taking fewer breaks, reporting screen flicker, and performing computed tomography screening were the principal predictors of increased eye strain symptoms in this study. Aronsson et al. [20] observed a significantly higher prevalence of eyestrain among radiologists compared to pediatricians. Two other studies carried out in Turkey included a sample, from two hospitals, of workers who used computers. The first [21] included secretaries, computer operators, and users of hospital data management systems. The second [15] did not specify whether or not they were health personnel. Azmoon et al. [22] concluded that defects in environmental conditions, such as thermal comfort and illumination intensity, increased eye fatigue in night shift nurses; however, the authors’ work was not aimed at the use of VDTs in the workplace and how it affects eye fatigue. Also, in 2019, a review [23] about CVS in radiologists concluded that long hours in front of computer monitors make the work of radiologists quite similar to that of computer professionals. 

Decision tree learning is one of the simplest yet most popular methods for inductive inference and optimization. It is able to obtain the best possible values of decision variables based on the selected objective function. In recent times, evolutionary algorithms have been widely used in various fields to find near-optimal solutions. 

The aim of this work is to identify the key factors that have the strongest influence on the prevalence of CVS in health workers. The results of an observational, cross-sectional, epidemiological study in the workforce at two public hospitals of Spain were used to develop a model based on regression trees and genetic algorithms. The performance of the model was tested in terms of its ability to predict the score achieved by an individual in the CVS questionnaire. The main findings may be a helpful guide for employers to design and implement more effective intervention programs.

This work is an extended version of a proceedings paper [24]. The increment includes an expanded introduction with further and updated references, a better description of the materials and of the algorithm developed, and a deeper and more structured discussion of the results.

## 2. Materials and Methods

### 2.1. Genetic Algorithms

The genetic algorithm (GA) is a well-known evolutive methodology, and one which is exceptionally useful for solving optimization problems. The basis of this method consists of the evolution of a population composed by a certain number of individuals [25]. The GA method was first proposed by Holland [26].

A GA always starts with the creation of a set of individuals that constitutes the first generation of the population. In the case of the present study, as in many others, the initial population is created at random, but taking into account that it would cover the whole solution space [27]. In this research, the GA is applied to a selection of variables, and therefore each member of the population is a bit string, where 1 means that the variable takes part in the model and 0 means that it does not. Previous research [28] has already employed this approach.

After setting the initial population, the main generational loop of the GA generates new offspring of candidate solutions by means of crossover and mutation operators, until the population is complete.

Another main GA operator is crossover, which combines the information of two or more members of the population [29]. In our case, each crossover is always performed on two different individuals. The mutation operator changes an individual by making random changes to it, using all the possible values that are present in the solution space. The use of the mutation operator makes it possible to create individuals that have some information that is completely different from that of the individuals of the previous generation. In some cases, this allows us to reach an optimum solution faster. In spite of this, and in order to guarantee the convergence of the method, it is advisable to maintain the probability of mutation at a relatively low value.

After the creation of a new population, it is evaluated with the help of the objective function. In order to allow the convergence of the algorithm, the best offspring solutions are selected, and thus they become the parents of the new generation.

Another operator that is used in the present research is called elitism. By means of this operator, some of the best solutions are transferred, with no changes from one generation to the next.

There must be a termination condition that defines when the algorithm terminates. In the case of the present research, a mechanism is additionally defined in order to stop the execution if, after a certain number of generations, the termination condition is not reached.

As in most evolutive algorithms, it must be remarked that the performance of GA is closely linked to the parameters employed, something that is connected to the nature of the problem under study. For the present research, and taking into account not only existing literature but also our own previous experience, the probability values applied for crossover were those from 0.5 to 1, in steps of 0.1, while the probability of mutation employed values were 0.1, 0.2, and 0.3. Finally, as stated before, relatively low values of elitism were employed: 0.01, 0.05, and 0.1, as in many other applications in existing literature [30].

### 2.2. Regression Trees

Regression trees are another well-known methodology, which analyse the relationships between variables with the help of a regression model with a tree-like structure [31] built by means of an iterative process. In recent years, regression trees have been successfully employed in different fields of knowledge, such as rock mechanics [32], electrical networks analysis [33], genome-wide association studies [34], or breast cancer analysis [35].

In a regression tree, the internal nodes are labelled with a set of mutually-exclusive conditions on the input variables $\left(x_{1},x_{2},\ldots ,x_{n}\right)$. For each condition, there is an associated edge that leads to a unique child node. All terminal nodes are linked to an estimation of the output variable (y). For each individual, a prediction is performed using the regression tree and assigning the input variable to any of the leaf nodes. In the present research, the standard binary regression tree is considered [36]. In this model, each bifurcation presents a condition related to only one input variable and with a single point estimate.

Regression trees are built in an iterative way that minimizes the residual squared error. This process is performed in a recursive way, until the termination condition is matched. In this case, either the residual error is zero, or the number of individuals is under certain a certain pre-determined value.

In general, a binary regression tree can be defined as a kind of tree that is built by means of successive recursive binary splits on variables of the form $x_{i}\leq c$ and $x_{i}\geq c$, where c∈R are observed values in a binary regression tree, expressed as

$$T\left(x\right)=\overset{M}{\underset{m=1}{\sum }}m\cdot B_{m}\left(x\right)$$

where T(x) represents the regression tree, M is the total number of terminal nodes of the tree, and $B_{m}\left(x\right)$ is the base function.

In our case, the basic function is defined as follows:

$$B_{m}\left(x\right)=\overset{L_{m}}{\underset{i=1}{\prod }}\left[x_{i}\left(m\right)-c_{im}\right]$$

where $L_{m}$ is the total number of splits, $x_{i}$ is the variable involved, and $c_{im}$ is the splitting value.

### 2.3. Study Population

To obtain the data, we performed an observational, cross-sectional, epidemiological study in two public hospitals in Spain: Monte Naranco Hospital (HMN) in Oviedo and the Central University Hospital of Asturias (HUCA). The former, which specializes in geriatrics and palliative care but also provides outpatient care and has surgical areas, started using EHR in 2007. The latter is the referral hospital for the Health Service of the Principality of Asturias, and started using EHR in 2014. The study included health personnel that were using VDTs at work in the following occupational groups: physicians and surgeons, residents, nurses, nurse specialists (including those in training), and nursing assistants.

Out of a total of 668 potentially eligible workers, 539 questionnaires were finally obtained (the response rate was therefore 80.69%). Of those, 196 were excluded, and so the final definitive sample of individuals that took part in the study was 343 workers (202 from HUCA and 141 from HMN), belonging to 47 different hospital units or departments. The reason for the exclusion of 66 workers was their suffering dry eye (35), amblyopia (6), squint (1), conjunctivitis (22), corneal ulcers (5), non-surgically controlled cataracts (5), glaucoma (1), blepharitis (2), keratitis (1), uveitis (1), vitreous disorders (8), and retinal disease (4). Another 116 were excluded because they did not use a computer for their job, and 13 were excluded because they had occupational groups outside the sampling criteria. Finally, one was excluded because of a lack of information on their seniority.

An informed consent form was signed by all the participants, in which data confidentiality was guaranteed during the entire process. The study was authorized by the Health Authority and approved both by the Research Ethics Committee of the Principality of Asturias and the Ethics Committee of the University of Alicante, Spain (the coordinator of the study), in accordance with the tenets of the Declaration of Helsinki.

### 2.4. Data Collection

The study was carried out between January and October 2017. Participants completed two self-administered questionnaires: the “Anamnesis and History of Exposure Questionnaire” and the “Computer Vision Syndrome Questionnaire” (CVS-Q).

The “Anamnesis and History of Exposure Questionnaire” was specifically developed for this study, in order to collect information about the personal and work characteristics of the subjects: gender, age, ophthalmic or contact lens use, history of eye disease and treatment, and any previous eye surgeries, as well as occupational groups (physicians and surgeons, including residents; nurses and nurse specialists, including those in training; and nursing assistants), work schedule (including morning shifts, evening shifts, rotating shifts without nights, and rotating shifts, including night and morning shifts plus on-call), hospital units or departments, seniority, perceived ease of use of the software, and daily VDT usage at and outside of work.

The “Computer Vision Syndrome Questionnaire (CVS-Q)”, designed and validated by Seguí et al. in 2015 [17], was used to measure perceived ocular and visual symptoms during or immediately following computer work. This questionnaire evaluates the frequency (never, occasionally, or often/always) and the intensity (moderate or intense) of 16 ocular and visual symptoms: burning, itching, sensation of a foreign body, tearing, excessive blinking, eye redness, eye pain, heavy eyelids, dryness, blurred vision, double vision, difficulty focusing for near vision, increased sensitivity to light, colored halos around objects, feeling that sight is worsening, and headache. Individuals with a score of 6 or more on the questionnaire are classified as symptomatic (suffering CVS).

### 2.5. The Proposed Algorithm

This study proposes a new algorithm that makes use of regression trees and GAs to assess the impact of the different variables on CVS.

**Figure 1:**
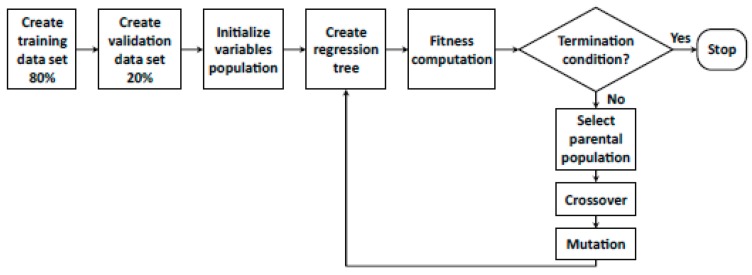
Algorithm diagram.

Due to the large number of variables available, GAs are employed in order to select subsets. As described in Figure 1, the first step of the algorithm involves the random selection of 80% of those individuals available on the database; in other words, the training data set is created. The remaining 20% of the information is later employed for the validation task.

The following stage of the algorithm is the creation of an initial population of those variables that will take part in the model.

Afterwards, regression trees are created and their performance evaluated by means of the root-mean-square error (RMSE). Until the termination condition is reached (RMSE not improved in more than 0.01% in the last 100 generations), many generations are created in the research of the subset of optimum variables.

One of the main drawbacks of the convergence of this method is that the fitness function is not evaluated for those individuals that are part of the training data set, but rather for those that form the validation data set. Although this makes the convergence lower, it makes the result obtained less generalizable.

Please note that in this work, and due to the total number of individuals available, instead of a *k*-fold cross-validation method, we have preferred to use 80% of the individuals for training and 20% for validation—but the algorithm was repeated a total of 1000 times. In other words, the algorithm was tested using 1000 different subsets for training and validation. Please note that by *k*-fold cross-validation, we refer to a methodology in which the original sample is randomly partitioned into *k* equal-sized subsamples. Of these, a single subsample is employed for validation, and the rest for training the model [37].

## 3. Results

### 3.1. General Characteristics of the Subjects

Table 1 summarizes the main characteristics of the sample. In terms of social demographic characteristics, the health workers were mainly women (77.3%), and their mean age was 46.9 ± 10.9 years, ranging from 22 to 67 years. Of the study population, 41.1% worked in the HMN hospital, and 60.3% of those in the geriatric department. This department is not available at the HUCA hospital, where 20.8% of the subjects worked in the pediatric department, and 18.3% in the medical and surgical hospital units. Only nursing staff (nurses and nursing assistants) were included in this category of medical and surgical hospital units. The average seniority in the occupational groups and in the hospital departments or units was 18.5 ± 11.5 years and 11.2 ± 11.0 years, respectively.

Most of the participants worked as a nurse (47.8%) and were ophthalmic lens wearers (70.6%), but only 15.5% were contact lens wearers. A total of 59.5% worked more than 4 hours per day with a computer, and 84% also used computers outside work. The average duration of VDT use at work was 5.1 ± 2.1 hours among men and 5.0 ± 2.2 hours among women. Men were also those who spent more time daily using a computer outside work (average duration 1.9 ± 1.3 hours) when compared to women (1.3 ± 1.1 hours).

The total number of workers who reported CVS was 195 (56.9%). Prevalence of CVS was significantly greater among contact lens wearers (75.5%) than non-wearers (53.4%) (*p* = 0.003). A significantly higher prevalence was also detected in ophthalmic lens wearers, and in those who use computers outside work (*p* < 0.05). 

### 3.2. Implementation of the Algorithm

**Figure 2:**
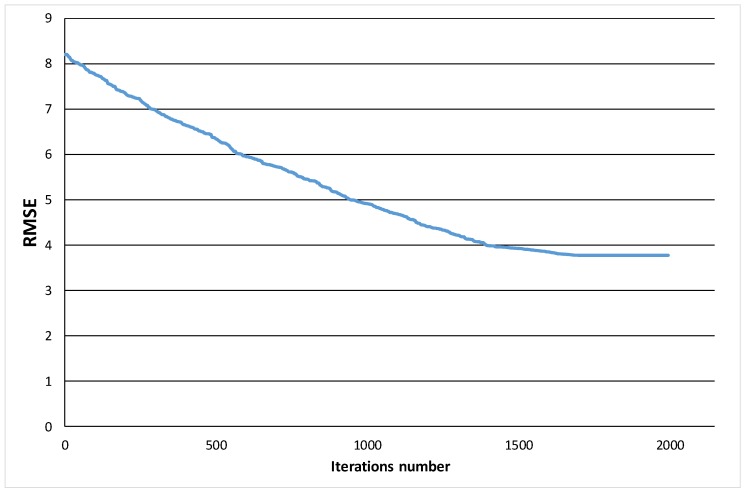
Evolution of the root-mean-square error (RMSE) value by the number of iterations.

The algorithm employed a total of 255 variables. As an example, Figure 2 shows how the RMSE changed as the number of iterations increased in one of the 1000 replicas performed. Please note that the best RMSE obtained in this case was 3.779221.

Only eight variables out of a total of 255 were included in more than 75% of the models, and 30 in more than 34%. Table 2 shows the variables that take part in most of the regression trees obtained, which are the most important to predict the score achieved by an individual in the CVS-Q: occupational seniority (97.20%), use of VDT at work (96.90%), hospital units’ seniority (96.30%), past history of conjunctivitis (88.50%), current use of eye drops (79.80%), working on rotating shifts including nights (74.60%), past history of refractive surgery (74.20%), and having been a VDT worker for more than two years (74.10%). 

**Figure 3:**
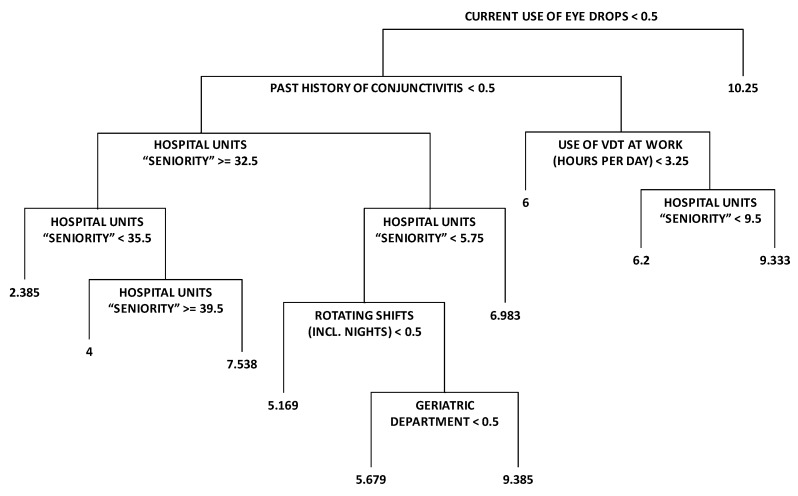
Example of regression tree obtained with the proposed algorithm.

**Figure 4:**
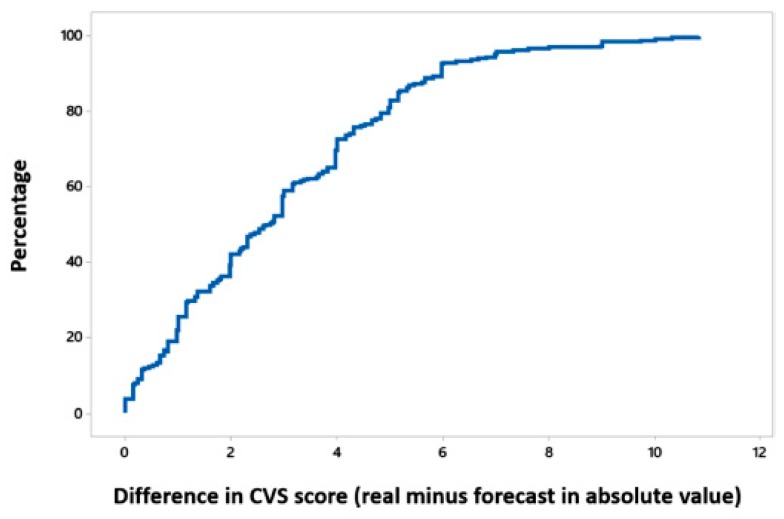
Cumulative distribution function of the difference in CVS score.

As an example, one of the regression trees generated is shown in Figure 3. The figures in the tree correspond to the average CVS score of the individuals classified in each node. In this case, the RMSE obtained was 3.740692. Figure 4 evaluates the model performance with the help of the cumulative distribution function of the difference in CVS score of real values and predictions. Please note how in 50% of the individuals, the absolute difference between their real CVS score and the forecasted value is below 3 points.

In order to determine how big the difference is between the real CVS values and their forecasts, Table 3 shows the average absolute differences. As can be observed, the smallest differences correspond to the CVS scores of 5, 6, and 7.

## 4. Discussion

Many methods can be implemented to analyze regression problems. Tree classification techniques are well-known for producing accurate predictions based on a relatively low number of if–then conditions. These techniques have been selected in the present research for precisely this reason. It is also important to point out that the results summarized in a tree are very simple for the user to interpret. Tree methods are nonparametric and nonlinear. Therefore, there is no implicit assumption that the underlying relationships between the predictor variables and the dependent variable are linear, follow some specific non-linear link function, or that they are even monotonic in nature.

The algorithm proposed in this work employs both regression trees and GAs, with the aim of finding better results than the regression tree itself. The GAs are employed to select those variables that would take part in the regression tree models. As is well-known, regression trees also select features by construction, so before implementing the present algorithm, regression trees were employed and their performance tested. In this case, the best RMSE value achieved was 4.804772, which is 27% higher than the one obtained with the proposed algorithm. This means that despite requiring a larger computational cost, the proposed algorithm is able to achieve a significantly better result.

As stated in the results, the minimum differences between the real CVS values and those forecasted by the model correspond to the CVS scores of 5, 6, and 7. This is a particular strength of the model, taking into account that the cutoff value to determine whether an individual suffers CVS is precisely 6.

According to the model, the most important variables for predicting the score achieved by an individual in the CVS-Q turned out to be occupational and hospital unit seniority, daily VDT usage at work and its duration, having suffered conjunctivitis in the past, current use of eye drops, working on rotating shifts (including nights) and on morning shifts plus on-call, a past history of ocular surgery (especially refractive surgery), being an ophthalmic or contact lens wearer, working in a geriatric department, and daily VDT usage outside work.

If the results achieved in the present study are compared with those obtained in a previous one performed by the authors [38], it may be observed that all the variables included in the previous model (gender, age, contact lens wearers, occupational category, work schedule, easy software application, use of VDT at work, use of VDT outside work, and current eye care), with the exception of current eye complaint, are listed in Table 2. The main difference between the two studies is that in the previous one, the model performed was only for classification (computer vision syndrome suffered or not), while the one developed in the present research calculates a score that predicts the one achieved by an individual in the CVS-Q.

The results of this study show that the total number of hours spent with a computer and occupational seniority are relevant variables in predicting the risk of CVS. This finding is in line with the study of Vertinsky et al. [19], which reported that the number of working hours had the strongest influence on eye strain in radiologists, especially when it is longer than six hours per day, and the risk could be decreased by taking breaks at least every hour. Other results also confirm that prolonged VDT use at work is linked to an increased risk of CVS [7,8], eyestrain [6,19], and other ocular and visual symptoms [39,40]. Moreover, dry eye disease is associated with prolonged VDT use of more than eight hours per day (OR, odds ratio = 1.94; 95% CI, 1.22–3.09) [41]. The relationship between the prevalence of CVS and greater occupational seniority is consistent with Ranasinghe et al. [7], who found out that the prevalence of CVS increased significantly (*p* < 0.01) with the number of years working in a job using a computer. Most of studies focus on the number of VDT use hours at work, but we have observed that the prevalence of CVS is higher among those who also use VDT outside of work, as it increases the total daily computer usage. In brief, the longer the daily exposure to VDT (at plus outside of work), the higher the proneness to suffer CVS.

According to the proposed model, CVS is also higher in workers who have suffered from conjunctivitis in the past, as well as in those who are using lubricant eye drops for dryness. Some diseases, such as allergic conjunctivitis, can be an extrinsic cause of evaporative dry eye. Thus, those workers might be affected by higher dryness. Dry eye was considered by Rosenfield et al. [4] to be a major contributor in the etiology of CVS, due to corneal drying from reduced blink rate and incomplete blinking. Nevertheless, they did not detect any evidence of a reduction in ocular and visual symptoms through the use of dry eye therapies, such as lubricating drops or rewetting drops.

Working in shifts, including nights, and to a lesser extent, working morning shifts plus on-call, was associated with the prevalence of CVS by the algorithm. Little is known to date about how shiftwork with VDT affects ocular and visual symptoms. Azmoon et al. [22] detected a relationship between thermal comfort, light intensity, sleep quality, and eye fatigue in night shift nurses. No reference is made to the use of VDT in their study. A consistent association of night-shift work with a higher risk for cardiovascular disease and cancer has been also reported [42,43]. In 2007, the International Agency for Research on Cancer classified night-shift work as a probable carcinogen, due to circadian disruption [44]. 

Scores of CVS are higher among those workers with a history of ocular surgery, and more especially refractive surgery. It is worth noting that laser surgery can change the shape of the cornea, and thus interfere in the relationship between the eyelids and ocular surface, disturbing normal blinking patterns [45].

Higher scores of CVS are predicted in ophthalmic and contact lens wearers. Several studies and reviews confirm that a regular use of contact lenses, particularly when worn for more than six hours of computer work, increases the prevalence of ocular and visual symptoms in daily computer users [4,8,16,23,38]. Nevertheless, none of this work detects any association between CVS and the use of ophthalmic lenses.

Health workers employed in a geriatric department tend to achieve higher CVS scores in the studied sample. A possible explanation for this is that in our study, all the workers in these units belonged to the HMN, where the EHR was introduced significantly earlier. This means that that hospital’s workers (including those in the geriatric department) have been exposed to VDT for much longer. This is consistent with those works previously mentioned, which confirm that the prevalence of CVS increases with the number of years working with a computer [7].

Nevertheless, given the limitations of our study, the results presented should be interpreted with caution. Firstly, it was a cross-sectional design, so it is not possible to ensure that the cause (use of VDT) precedes the effect (presence of CVS). Secondly, no ophthalmic examinations were performed to determine the workers’ refractive state. Finally, we only took into consideration the use of computers at and outside work, but not the use of mobile or other hand-held devices, which could be a confounding factor. 

Despite these limitations, a particular strength of this work lies in its use of a validated questionnaire to measure CVS. This study develops the first model that predicts the score achieved by an individual in the CVS-Q. Also, it is one of the few studies that include healthcare workers. Finally, it is important to remark that the main contribution of the present research is a novel methodology, able to determine the most important variables in a prediction problem. We would like also to highlight that from a machine learning point of view, 343 questionnaires do not represent a huge amount of information, and the performance of this method with large data sets should be examined in greater depth.

## 5. Conclusions

In summary, this epidemiologic study in Spain has been able to construct an algorithm for CVS prediction, using only data that can be obtained by two simple self-administered questionnaires that were designed to be easily completed by workers when they are undergoing eye examinations. By combining the capabilities of a genetic algorithm and regression trees, this model is able to determine the most important variables that influence CVS, and predict the score achieved by the subject in the CVS-Q.

The findings of the present investigation confirm a high prevalence of CVS in a sample of healthcare workers at two public hospitals, and associate it with longer duration of occupation and higher daily computer usage. These results are similar to those of other studies that focused on different populations (office workers, bank employees, high-tech workers, among others). Long duration of VDT use makes the work of physicians, surgeons, and nursing staff quite similar to these other occupations in this respect, so a key conclusion is the importance of studying CVS among health personnel. Further larger-scale studies are needed to address the effect of VDT use on CVS in health personnel workforce, and provide guidance regarding good working practices to minimize the severity of these ocular symptoms.

## Figures and Tables

**Table 1:** Characteristics of the sample and prevalence of Computer Vision Syndrome (CVS).

Variables	No. of Subjects (*n* = 343)	No. with CVS (*n* = 195)	*p*-Value
Sex			
male	78 (22.7%)	37 (47.4%)	0.056
female	265 (77.3%)	158 (59.6%)	
Age (years)			
≤30	32 (9.3%)	16 (50.0%)	0.062
31–40	73 (21.3%)	42 (57.5%)	
41–50	93 (27.1%)	62 (66.7%)	
51–60	107 (31.2%)	60 (56.1%)	
>60	38 (11.1%)	15 (39.5%)	
Hospital			
HMN	141 (41.1%)	85 (60.3%)	0.284
HUCA	202 (58.9%)	110 (54.5%)	
Ophthalmic lens wearers			
no	101 (29.4%)	49 (48.5%)	0.044
yes	242 (70.6%)	146 (60.3%)	
Contact lens wearers			
no	290 (84.5%)	155 (53.4%)	0.003
yes	53 (15.5%)	40 (75.5%)	
Ocular Surgery			
no	309 (90.1%)	173 (56.0%)	0.330
yes	34 (9.9%)	22 (64.7%)	
Occupational groups			
physicians and surgeons, including residents	128 (37.3%)	63 (49.2%)	0.056
nurses and nurse specialists, including those in training	164 (47.8%)	104 (63.4%)	
nursing assistants	51 (14.9%)	28 (54.9%)	
Work schedule			
morning shifts	133 (38.8%)	71 (53.4%)	0.075
evening shifts	3 (0.9%)	0 (0.0%)	
rotating shifts, without nights	24 (7.0%)	14 (58.3%)	
rotating shifts, including nights	100 (29.2%)	66 (66.0%)	
morning shifts plus on-call	83 (24.2%)	44 (53.0%	
Easy software application			
no	74 (21.6%)	39 (52.7%)	0.416
yes	269 (78.4%)	156 (58.0%)	
Use of visual display terminals (VDT) at work (hour per day)			
<2	27 (7.9%)	13 (48.1%)	0.534
2–4	112 (32.7%)	67 (59.8%)	
>4	204 (59.5%)	115 (56.4%)	
Use of computer outside work			
no	55 (16.0%)	24 (43.6%)	0.031
yes	288 (84.0%)	171 (59.4%)	

**Table 2:** Percentage of regression trees in which each variable is included.

Variables	Percentage of Models
Occupational seniority (years)	97.20%
Use of VDT at work (hours per day)	96.90%
Hospital units’ seniority (years)	96.30%
Past history of conjunctivitis	88.50%
Current use of eye drops	79.80%
Rotating Shifts (including nights)	74.60%
Refractive surgery	74.20%
Time as VDT worker (>2 years)	74.10%
Ocular surgery	69.40%
Ophthalmic lens wearers	64.10%
Geriatric department	60.70%
Use of VDT outside work (hours per day)	60.60%
Total VDT use (hours per day)	60.10%
Morning shifts plus on-call	57.20%
Sterilization unit	53.40%
Contact lens wearers	50.30%
Surgery unit	48.60%
Past history of ocular herpes	46.10%
Age	42.40%
Blood bank department	41.10%
Anatomical pathology department	40.90%
Endocrinology unit	40.70%
Morning shifts	40.50%
Sex	40.20%
Traumatology unit	39.90%
Nephrology unit	39.70%
Easy software application	39.60%
Past history of keratitis	39.30%
Anesthesiology department	34.60%
Evening shifts	34.20%

**Table 3:** Average differences in absolute value of the real CVS value and the forecast.

CVS Value	Avg. Difference	*n*
0	5.224	19
1	4.356	24
2	3.855	16
3	3.558	17
4	2.351	38
5	1.587	34
6	1.21	27
7	0.991	27
8	1.671	31
9	2.313	20
10	2.499	24
11	3.27	21
12	3.899	14
13	5.647	8
14	5.204	8
15	6.127	3
16	9.386	7
17	8.816	2
19	9.667	1
20	9.75	1
**All**	**2.976**	**343**
